# 

**Published:** 1910-10-15

**Authors:** 


### THE HOSPITAL

Telegraphic Address: Ospedale, London.

**THE MODERN MEDICAL AND INSTITUTIONAL JOURNAL.**

Telephone Number: 2734 Gerrard.

**NEW SERIES. No. 192, Vol. VIII. No. 1264, Vol. XLIX.** SATURDAY, OCT. 15TH, 1910. **PRICE THREEPENCE**

